# A Multi-Attribute Decision-Making Model for the Selection of Polymer-Based Biomaterial for Orthopedic Industrial Applications

**DOI:** 10.3390/polym14051020

**Published:** 2022-03-03

**Authors:** Ali Rizwan, Emad H. Abualsauod, Asem Majed Othman, Suhail H. Serbaya, Muhammad Atif Shahzad, Abdul Zubar Hameed

**Affiliations:** 1 Department of Industrial Engineering, Faculty of Engineering, King Abdulaziz University, Jeddah 21589, Saudi Arabia; sserbaya@kau.edu.sa (S.H.S.); mmoshtaq@kau.edu.sa (M.A.S.); aahameed@kau.edu.sa (A.Z.H.); 2 Department of Industrial Engineering, College of Engineering, Taibah University, Medina 41411, Saudi Arabia; eabualsauod@taibahu.edu.sa; 3 Department of Industrial and Systems Engineering, College of Engineering, University of Jeddah, Jeddah 21959, Saudi Arabia; amothman@uj.edu.sa

**Keywords:** chemo metric analysis, UHMWPE, FTIR spectroscopy, industrial applications, MWCNTs, PCA, optimization, scree plot

## Abstract

The potential of quantifying the variations in IR active bands was explored while using the chemometric analysis of FTIR spectra for selecting orthopedic biomaterial of industrial scale i.e., ultra-high molecular weight PE (UHMWPE). The nano composites UHMWPE with multi-walled carbon nano-tubes (MWCNTs) and Mg-silicate were prepared and irradiated with 25 kGy and 50 kGy of gamma dose. Principal component analysis (PCA) revealed that first three principal components (PCs) are responsible for explaining the >99% of variance in FTIR data of UHMWPE on addition of fillers and/or irradiation. The factor loadings plots revealed that PC-1 was responsible for explaining the variance in polyethylene characteristics bands and the IR active region induced by fillers i.e., 440 cm^−1^, 456 cm^−1^, from 900–1200 cm^−1^, 1210 cm^−1^, 1596 cm^−1^, PC-2 was responsible for explaining the variance in spectra due to radiation-induced oxidation and cross linking, while the PC-3 is responsible for explaining the variance induced because of IR active bands of MWCNTs. Hierarchy cluster analysis (HCA) was employed to classify the samples into four clusters with respect to similarity in their IR active bands which is further confirmed by PCA. According to multi attribute analysis with PCA and HCA, 65 kGy irradiated sample is optimum choice from the existing alternatives in the group of irradiated pristine UHMWPE, UHMWPE/Mg-silicate irradiated with 25 kGy of gamma dose was the optimum choice for UHWMPE/Mg-silicate nano composites, and UHMWPE/γMWCNTs composites containing 1.0% dof γ MWCNTs for UHMWPE/MWCNTs nanocomposites, respectively. The results show the effectiveness of quantifying the variance for decision as far as optimization of biomaterials in orthopedic industrial applications is concerned.

## 1. Introduction

Ultra-high molecular weight PE (UHMWPE) is the gold standard material for orthopedic industrial applications from last ten decades owing to its various properties including biocompatibility, high wear resistance, low friction coefficient, suitable stiffness, and fatigue resistance [1,2,3,4]. However, the osteolysis and aseptic loosening are the critical disadvantages which limit the service life of prosthesis. To cope with the limitations, the process of cross linking or irradiation of UHMWPE is used in highly cross-linked polyethylene [1,4,5,6,7,8], but the main interest continues to refer the long-term oxidation stability of highly cross-linked UHMWPE. This is due to the formation of free radicals during the process of irradiation, which are responsible for continuing long-term oxidation reactions thus limiting the service life of UHMWPE. In order to stabilize the radiation-induced free radicals, number of methodologies are adopted including post irradiation annealing, melting, incorporation of bio-compatible of anti-oxidants, i.e., α-tocopherol (vitamin E) [9,10,11], irradiating the UHMWPE in the presence of predetermined amount of organo-silane [6,12,13,14,15], using the suitable silane-based clay [16], and preparing the UHMWPE nano composites with MWCNTs for quenching the radiation-induced free radicals [7,8,17,18].

Among the aforementioned latest alternative approaches, nano-composites of UHMWPE based on carbon nanotubes (CNT) have acquired enormous concern due to their high chemical stability as well as improved properties, principally mechanical behavior, thermal and optical properties, and electrical conductivities [19]. Owing to exceptional characteristics, CNT-reinforced polymeric composites have remained the center of interest for researchers [20]. There have been reports of limited successful outcomes for UHMWPE reinforced with MWCNTs [18,21,22]. Although, there is still a disagreement related to the biocompatibility of CNT at the present time, because some researchers investigated their cytotoxicity [23,24,25], while other authors reported that CNTs are considered as perfect substrates for cellular development [26,27].However, it is a well-accepted fact that CNTs and multi-walled CNTs with multi-walls i.e., MWCNTs have induced an intense interest for a number of industrial applications including the biomaterials, sensors, catalyst supports, and energy storage devices mainly because of their physical and mechanical characteristics [28,29,30]. It has been described in literature that significant features for the characterization of MWCNTs are surface properties, imperfection sites, and graphitization [31].Numerous techniques are employed in order to achieve MWCNTs with optimized properties. These techniques include treating them with chemicals, ultrasonic waves, mechanically, and with energetic radiations [31,32]. It has been revealed in modern research that treating CNTs with high-energy radiations such as gamma, e-beam, and ions can be used for inducing the molecular connections between the polymers and CNTs. Furtherance to it, irradiations can also be used for fictionalization of CNTs while inducing the functional groups at their surfaces thus providing a new direction to improve the CNTs’ utility and characteristics [33].

Among the radiations, gamma ray has drawn full attention for the reason that it is considered for the optimization method to improve the characteristics (chemical and physical) of MWCNTs. It has been reported that treating MWCNTs with gamma rays in the presence of dilute sulfuric acid results in shortening of the MWCNTs. Gamma radiations are also used for improving the surface functioning of the carbon fiber (CF) [33,34,35]. Furthermore, gamma radiations are also effective for increasing the cross linking yields with the polymer matrix while inducing the intense interaction between the polymer chains and CNTs. It has been demonstrated in the literature [6,36,37] that by applying gamma ray irradiation, the mechanical and electrical characteristics of polymer nano composites can be revised. On the other hand, literature reported that treating MWCNTs with gamma radiation in air is responsible for reducing the distance (inter wall) of MWCNTs. This reduction in inter wall distances is responsible for updating the graphitic order of MWCNTs. However, irradiating MWCNTs in epoxy chloropropane (ECP) results in increasing the inter wall distance of MWCNTs and is responsible for the loss of graphitic order. Moreover, gamma rays are also responsible for enhancing the hydrogen adsorption capacity of MWCNTs and the amount of functional-groups attached to the surface of MWCNTs. This is because of an increase in imperfections at MWCNTs surface by gamma radiations [38,39,40].

The main theme of this paper is to explore the efficacy of MWCNTs (unmodified and gamma modified) for quenching the radiation-induced free radicals when UHMWPE is treated with gamma sterilization doses in the presence of MWCNTs (unmodified and gamma modified). Therefore, UHMWPE composites with MWCNTs and gamma-irradiated MWCNTs (γMWCNT) that is UHMWPE/γ-MWCNT and UHMWPE/MWCNTs were prepared with 0.5% and 1.0% of fillers by wt. Subsequently, the prepared composites were treated with gamma doses of 25 kGy and 50 kGy. After irradiations, FTIR spectroscopy with chemo metric analysis was employed to probe the useful aspects of radiation-induced defects and/or functional groups at the surface of MWCNTs. The chemo metric analysis was performed while using multivariate statistical techniques including the hierarchy cluster analysis, principal component analysis for identification of the main source of variances for both types of carbon-based composites i.e., UHMWPE/MWCNTs, UHMWPE/γ-MWCNTS. Furtherance to it, the results were also compared with surface-functionalized UHMWPE composites with Mg-silicate, a recently proposed hybrid clay filler.

## 2. Material and Methods

### 2.1. UHMWPE/MWCNTs Composites

#### 2.1.1. Preparations

During the course of this study following chemicals and materials (purchased from Sigma Aldrich, Burlington, MA, USA) were used:

- UHMWPE resin powder having density d = 0.940 g cm^−3^
- Un-irradiated and γ-irradiated MWCNTs
- 99% pure acetone and methanol.

The chemicals were used without further treatment/purification. The appropriate concentrations (0.5% and 1.0%) of un-irradiated and gamma-treated MWCNTs were mixed in 100 mL of methanol. The mixture was then sonicated in Elma Sonic E 30 ultra-sonic bath (Elma Schmidbauer GmbH, Singen, Germany). for 45–50 min to disperse MWCNTs (un-irradiated and gamma irradiated) in methanol. Afterward the solution was mixed well with UHMWPE resin powder with the help of a glass rod to obtain the homogenized mixture of UHMWPE and MWCNTs. The admixture was then put in the oven at 50 °C for 24 h to evaporate methanol. After the preparation of mixture, the sheets of micron size were prepared following the well-documented compression molding protocol of UHMWPE and its composites [36,37,41,42]. The UHMWPE/Mg-silicate samples were prepared while following methodology as reported by other researchers [16].

#### 2.1.2. Irradiation and Labeling

After preparation of the sheets of UHMWPE composites (with MWCNTs and Mg-silicate), three groups of samples was made, the control samples were kept at shelf and categorized and others were sent for radiation treatment. The irradiation treatment was done in open air at 25 °C with 25, 50 kGy of gamma dose. The ^60^Co source was used for radiation treatment. After the radiation treatment, samples were labeled with specific codes. These codes were used for identification purpose and reflect the nature of fillers (un-irradiated MWCNTs and gamma irradiated MWCNTs) along with their concentrations. The sample codes are given in Table 1. Pure UHMWPE is irradiated with five dose values ranging from 25–100 kGy, respectively.

Note the sample code

- “P” represents the UHMWPE without any filler;
- “P-γCNTs” represent the UHMWPE composite with gamma irradiated MWCNTs;
- “P-CNTs” represent the UHMWPE composite with MWCNTs;
- “P-SP” represents the UHMWPE composite with Mg-silicate.

It is worth to mentioned here that the subscript denotes the concentration of fillers and superscript denotes the value of gamma dose e.g., P-γ ${\mathrm{CNT}}_{0.5}^{50}$ stands for the UHMWPE composite containing 0.5% γMWCNTs (by wt.) and treated with 50 kGy of gamma dose.

### 2.2. Structural Characterization

#### 2.2.1. Fourier Transform Infrared (FT-IR) Analysis

The FTIR spectra of γ-MWCNTs and UHMWPE/γ-MWCNTs were recorded in total attenuated reflectance mode using Thermo-Nicolet 6700 (Thermo Fisher Scientific, Waltham, MA, USA) Fourier transform infrared spectrophotometer (schematic diagram shown in Section 3.1) from 4000–400 cm^−1^ at a resolution of 6 cm^−1^. The spectra were collected after acquiring 216 scans. In order to reduce the signal to noise (S/N) ratio, spectra of composites were taken from three/four different positions of each sample and then averaged.

#### 2.2.2. Chemometric Analysis

Subsequent to experimental method, multivariate statistical analysis of all the registered spectra has been performed. The analysis included the hierarchy cluster analysis (HCA) and principal component analysis (PCA). HCA arranged all the samples into four clusters on the similarity of IR active bands while PCA unveiled the hidden factors responsible for arranging the samples into the four clusters by HCA.

## 3. Results and Discussions

### 3.1. FTR Analysis

The significant IR active bands that are strongly affected either by incorporation of fillers and/or by gamma treatment are enlisted in Table 2. The potential of fillers i.e., MWCNTs and γ-MWCNTs and Mg-silicate and/or gamma doses for UHMWPE structural improvement can be revealed by investigating the enlisted IR active bands. There are significant updates in the FTIR spectra of UHMWPE upon addition of fillers i.e., MWCNTs and γ-MWCNTs and Mg-silicate and/or gamma treatment. Some of the significant updates are:

- Alteration in C–H bending and stretching (asymmetric and symmetric) absorption;
- Appearance of COO–, C–O stretching and C–C stretching absorption bands at 1596 cm^−1^, 1262 cm^−1^ and in the range of 1100–1000 cm^−1^, respectively;
- Appearance of absorption peaks because of carbonyl products (–C=O);
- Update in different stretching, contracting, and bending of PE chain (–CH_2_) because of addition of fillers and/or gamma irradiations;
- Appearance of absorption peaks because of some end products of peroxides such as ketones, and acids etc.

**Figure 1:**
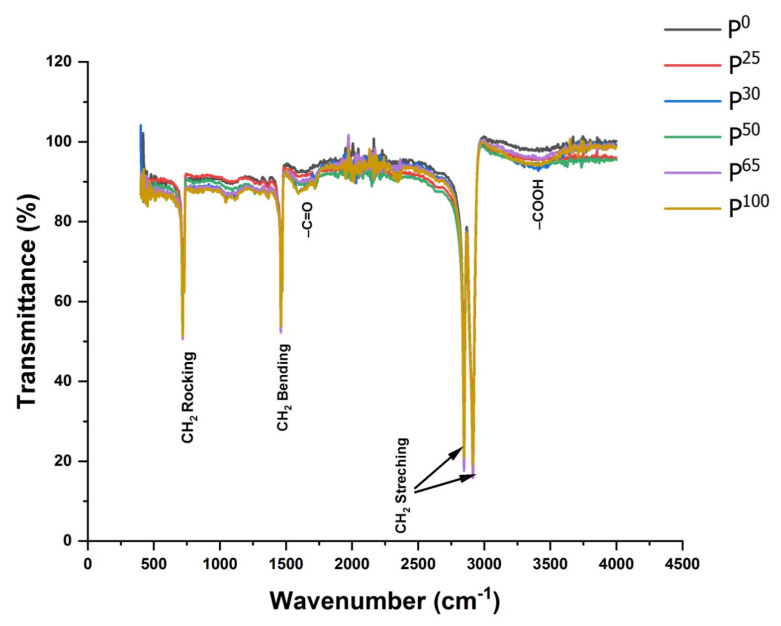
UHMWPE samples (un-irradiated and gamma-treated 25–100 kGy) FT-IR spectra.

For investigating the effect of gamma irradiations alone, FTIR spectra of UHMWPE samples irradiated with gamma dose ranging from 25–100 kGy are shown in Figure 1, while the IR active bands of interest for this particular set of group are represented in Table 2 in 1st column labeled as polyethylene. The polyethylene characteristics of IR bands i.e., –CH_2_ stretching vibrations at 2849 and 2924 cm^−1^, –CH_2_ bending vibrations at 1460 and 1470 cm^−1^, and rocking-deformation belonging to –CH_2_ long chain at 717 and 730 cm^−1^ are evident for all spectra. A more analytical view of this set of spectra revealed the modifications in region 1450–1480 cm^−1^, 1650–1850 cm^−1^, 2800–2950 cm^−1^, and 3000–3750 cm^−1^. These regions belong to –CH_2_ bending vibrations, absorption due –CH_2_ units in amorphous region, –C=O absorptions, –CH_2_ stretching vibrations, and peroxides bonded regions, respectively. These modifications in IR spectra on gamma irradiation are basically due to the crosslinking and/or post crosslinking oxidation of UHMWPE. For the sample with 0 kGy of gamma dose, absorption due to –C=O in the region 1650–1850 cm^−1^ is almost negligible and changes in area belonging to peroxide bounded products i.e., 3000–3750 cm^−1^ are almost zero, showing that there is no oxidation in this sample. However, for irradiated samples, situation is quite different because samples suffer many chemical and physical changes because of radiation-induced free radicals. These free radicals act in various ways, e.g., they can react to form crosslinks, further breakage of PE chains, and to react with diffused oxygen with the polyethylene matrix following the well-established oxidation chain reactions of polyethylene [39,40,41,42,43]. Furthermore, the amount of free radicals is linearly correlated with the absorbed gamma dose, therefore, modifications in all above-mentioned regions, i.e., –CH_2_ bending vibrations, absorption due –CH_2_ units in amorphous region, –C=O absorptions, –CH_2_ stretching vibrations, and peroxides bonded regions are higher for 65 kGy and 100 kGy.

**Figure 2:**
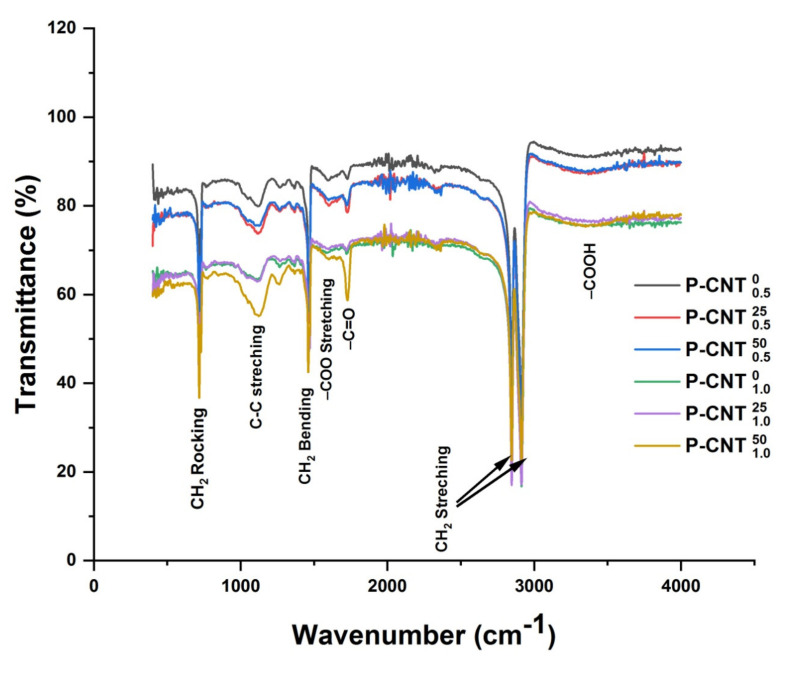
FTIR spectra of UHMWPE/MWCNTs with 0.5 and 1.0% of MWCNTs, and treated with gammadose of 25 and 50 kGy, respectively.

For quantifying the effect of addition of MWCNTs and irradiating UHMWPE in the presence of MWCNTs, FTIR spectra of the UHMWPE/MWCNTs nano composites containing 0.5% and 1.0% of MWCNTs are shown in Figure 2. These composites were irradiated with 25 kGy and 50 kGy of gamma doses, respectively. The IR active bands of particular interest for this group are tabulated in Table 2 in 2nd column labeled as UHMWPE nano composites. The characteristics bands of polyethylene are evident in all spectra. In addition to polyethylene, characteristics bands following significant modifications are evident on addition of MWCNTs in UHMWPE and irradiating it with 25 and 50 kGy of gamma dose.

- Increase in absorbance for whole spectral range i.e., 400–4000 cm^−1^ on addition of MWCNTs, and this increase in absorbance is approximately 6% for composites having 0.5% of MWCNTs and 18% for composites containing 1.0% of MWCNTs.
- On addition of MWCNTs, appearance of absorption in region 900–1200 cm^−1^ with peak absorbance at 1100 cm^−1^ is quite evident. This peak belongs to C–C stretching absorption and it further increases with the concentration of MWCNTs and absorbed dose. The highest absorption in this region is observed for UHMWPE/MWCNTs nano composites containing 1.0% MWCNTs (by wt.) and irradiated with 50 kGy of gamma dose.
- On addition of MWCNTs and further irradiation, appearance of additional peak at 1262 cm^−1^ is observed which belongs to C–O stretching absorption. The trend of this peak is similar as that of C–C stretching absorption with respect to concentration of MWCNTs and gamma dose.
- Another peak at 1596 cm^−1^ is also observed for all samples on addition of MWCNTs. This peak belongs to –COO stretching absorption and follows the same trend as earlier.
- A significant increase in absorption in the region 1600–1800 cm^−1^ having peak absorbance at 1718 cm^−1^ is observed on irradiating the samples with gamma dose. The absorbance is higher for composite containing 1.0% of MWCNTs and irradiated with 50 kGy of gamma dose. This absorbance is due to C=O functional group and is used for assessing the degradation of polyethylene because of free radicals-induced oxidation chain reactions.
- In addition to this, polyethylene characteristics bands, i.e., –CH_2_ stretching, rocking, and bending vibrations also suffer modifications because of the addition of MWCNTs and irradiations.

**Figure 3:**
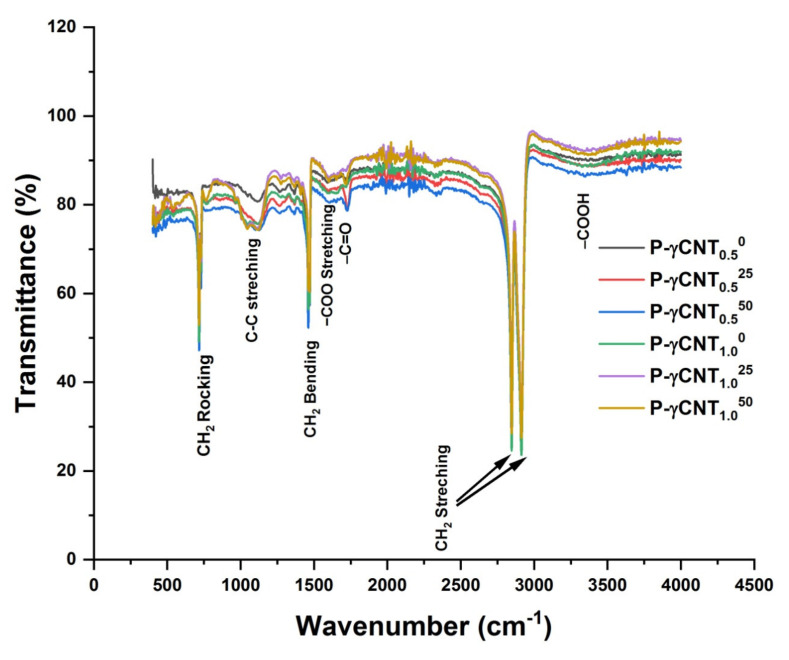
FTIR spectra of UHMWPE/γMWCNTs with 0.5% and 1.0% of γ-MWCNTs, and treated with gammadose of 25 and 50 kGy, respectively.

Now coming to UHMWPE, nano composites with 0.5% and 1.0% (by wt.) of gamma ray modified MWCNTs, i.e., UHMWPE/γMWCNTs composites. The modifications and/or appearance of additional IR absorption peaks which are mentioned above (for the case of UHMWPE/MWCNTs nano composites) are similar, i.e., appearance of additional absorption peaks due to C–C stretching, C–O stretching, and –COO stretching at 1100, 1262, and 1595 cm^−1^ are evident in Figure 3. However, FTIR spectra of UHMWPE/γMWCNTs are quite different from UHMWPE/MWCNTs on further irradiation with gamma dose. A few notable differences of particular as far as subject matter of interest is concerned are:

- The increase of absorbance over the whole spectral range, i.e., from 400–4000 cm^−1^ for all UHMWPE/γMWCNTs samples is approximately 7% as opposite to UHMWPE/MWCNTs composites where the increase for absorbance is 18% for sample containing 1.0% of MWCNTs (see Figure 2).
- Most importantly, there is a significant reduction in absorbance for carbonyl-associated region, i.e., 1650–1850 cm^−1^ on further irradiating the composites with gamma dose. This reduction is higher for UHMWPE/γMWCNTs composite containing 1.0% of γMWCNTs and irradiated with 50 kGy of gamma dose thus revealing the free radical quenching potential of γMWCNTs.
- The absorbance due to C–C stretching absorption (from 900–1200 cm^−1^) is higher for UHMWPE/γMWCNTs composite containing 1.0% of γMWCNTs and irradiated with 50 kGy of gamma dose and seems to be negatively correlated with absorbance of carbonyl-associated region i.e., 1650–1850 cm^−1^.

The presence of defective sites within MWCNTs and the role of gamma irradiation for enhancing the structural quality of MWCNTs for γMWCNTs can be used here to explain the difference between the two sets of groups. It is well established that γ-irradiation of MWCNTs with a dose value of ≤100 kGy improves their quality by eliminating the already present defective sites within MWCNTs matrix [44], and irradiating in open air is also responsible for inducing functional groups at the surface of MWCNTs. These are –C=O, –COO, –C–O, –C–C–, and –C=C– containing functional groups, and have the potential of neutralizing the radiation-induced free radicals while reacting with primary free radicals [33,44,45]. The reduction of absorbance belonging to polyethylene oxidation peak i.e., from 1650–1850 cm^−1^ might be due to the aforementioned reason. The increase in absorbance belonging to C–C stretching vibration for UHMWPE/γMWCNTs nano composites containing 1.0% of fillers is also in favor of argument about the efficacy of radiation-induced functional groups (at the surface of γMWCNTs) in neutralizing the polyethylene free radicals. In addition to this the elimination of defective sites upon irradiating MWCNTs with a dose value of ≤100 kGy is responsible for smaller absorbance by UHMWPE/γMWCNTs composites over the whole spectral range as compared to UHMWPE/MWCNTs composites as shown in Figure 2 and Figure 3.

**Figure 4:**
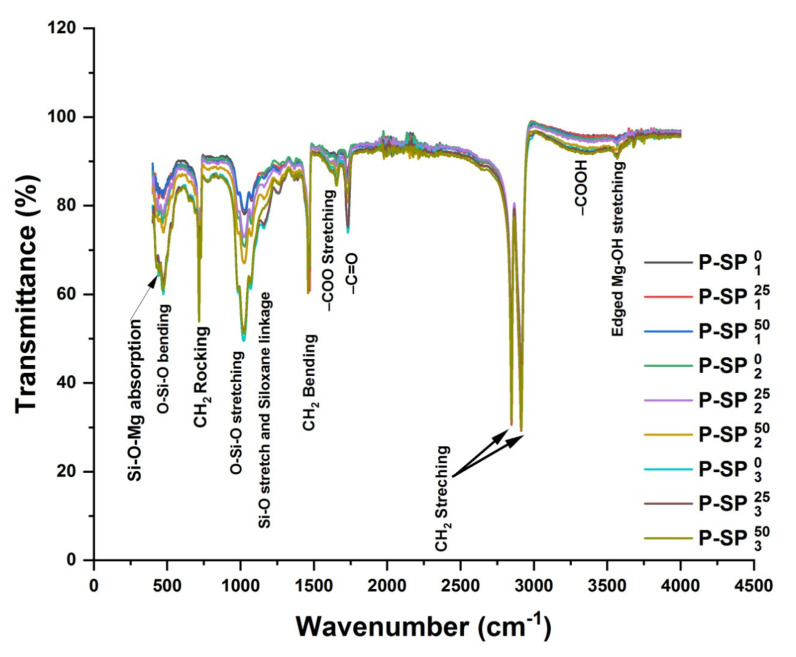
UHMWPE/Mg-silicate FT-IR spectra containing 1.0%, 2.0%,and3.0% (by wt.) fillers, and treated with gammadose of 25 and50 kGy, respectively.

Figure 4 represents the IR spectra of UHMWPE/Mg-silicate nano composites with 1.0%, 2.0%, and 3.0% of Mg-silicate by wt. and irradiated with 25 and 50 kGy of gamma dose. It can be seen from the figure that absorption of the C=O functional group is found to increase for composites and this increase is dependent on the amount of filler as well as absorbed dose. A significant increase in absorption is also found for characteristics poly1zethylene bands i.e., CH_2_ bending, stretching, and rocking deformations, respectively. In addition to this, modifications absorption in polyethylene crystalline as well as amorphous bands i.e., at 1896 cm^−1^ and 1305 cm^−1^ are also evident from the FTIR spectra given in Figure 4. All the aforementioned alterations in polyethylene characteristics and oxidation degradation bands are dependent on Mg-silicate as well as absorbed dose. However, further irradiating the composites with 25 and 50 kGy of gamma dose results in an increase of absorbance for characteristics bands associated with Mg-silicate i.e., O–Si–O stretching, Si–O stretching, and siloxane linkage absorption, which is small for P-SP1, adequate for P-SP2, and negligible for P-SP3, as evident from the insets of the figure. Furthermore, increase in absorption under the peroxide-bonded region and edged Mg–OH stretching vibration peak is also observed which is higher for P-SP3 samples irradiated with 25 and 50 kGy of gamma dose.

### 3.2. Chemometrics Studies

#### 3.2.1. Hierarchy Cluster Analysis (HCA)

**Figure 5:**
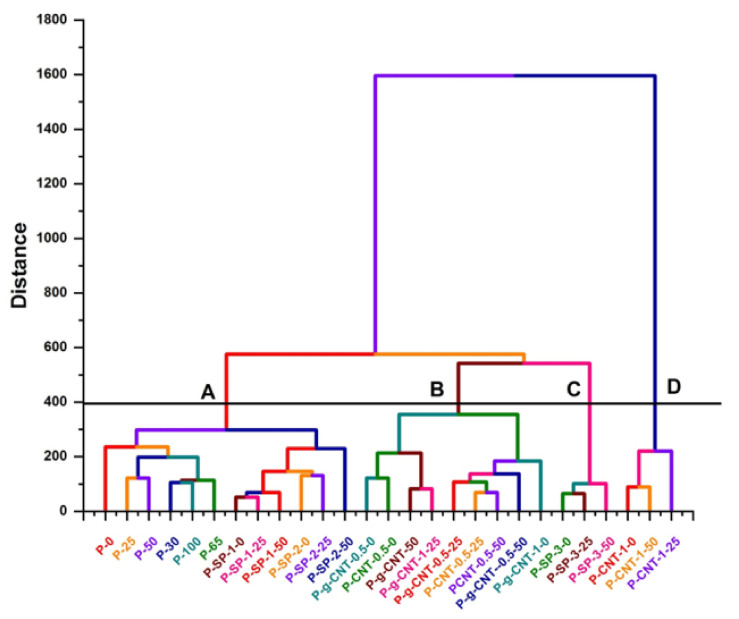
Dendrogram obtained from hierarchy cluster analysis (HCA) representing the variation of samples from reference, i.e., UHMWPE upon addition of fillers and gamma irradiations. Note this dendrogram is generated on the basis of dissimilarities in FTIR spectra of samples.

The organization of existing alternatives in clusters of similar characteristics is performed while using hierarchy cluster analysis (HCA) and the results are represented in the form of a dendrogram as a function of Euclidian distance from reference, as shown in Figure 5. The HCA arranged all the samples in four clusters A, B, C, and D, where groups A and B are further divided into two subgroups A_1_ and A_2_, and B_1_ and B_2_, respectively. All the samples are arranged on the relative scale on the basis of variations in IR active bands associated within each set of samples or group. For example, sub group A_1_ consists of pristine UHMWPE sample irradiated with gamma dose ranging from 25–100 kGy, and it can be seen from the figure that sample UHMWPE irradiated with 65 kGy has larger variance in IR active bands, this cluster is based only on the characteristics polyethylene bands. The sub group A_2_ consists of UHMWPE/Mg-silicate composites with 1.0% and 2.0% of Mg-silicate by wt. The Mg-silicate IR active bands along with polyethylene characteristics ones are major liable factors for making this cluster. The UHMWPE/Mg-silicate composite containing 3.0% of Mg-silicate form the cluster labeled as C. The position of P–SP_3_^50^ relative scale confirms that this composite suffers more when irradiated with 50 kGy of gamma dose as evident from FTIR data of UHMWPE/Mg-silicate (see Figure 4). The groups B and D are the clusters containing UHMWPE composites with γMWCNTs and MWCNTs, respectively. A close quantitative look while considering the amount of variations with reference IR active bands i.e., UHMWPE, composites with MWCNTs i.e., members of group D show larger variations because of defective sites within the MWCNTs. In short, the HCA gives an idea about the suitable choice for UHMWPE for orthopedic medical applications on relative scale. As it is required for such applications that structural variations should be lower along with lower value of Euclidian distance, therefore, 65 and 100 kGy irradiated ones are the reasonable choice for irradiated pure UHMWPE, and for UHMWPE/Mg-silicate composites the optimum choice is UHMWPE/Mg-silicate composite containing 1.0% of Mg-silicate as compared to composites containing 2.0% and 3.0% of Mg-silicate. From UHMWPE composites with MWCNTs, using the gamma irradiate composites is beneficial as compared to un-irradiated ones. However, for unveiling a few important questions like which variations in IR active bands needs to be considered and how the variances of various IR active bands in clusters could be quantified with respect to the shift in absorption intensities, FTIR data are further analyzed with PCA and the results are presented below.

#### 3.2.2. Principal Component Analysis (PCA)

**Figure 6:**
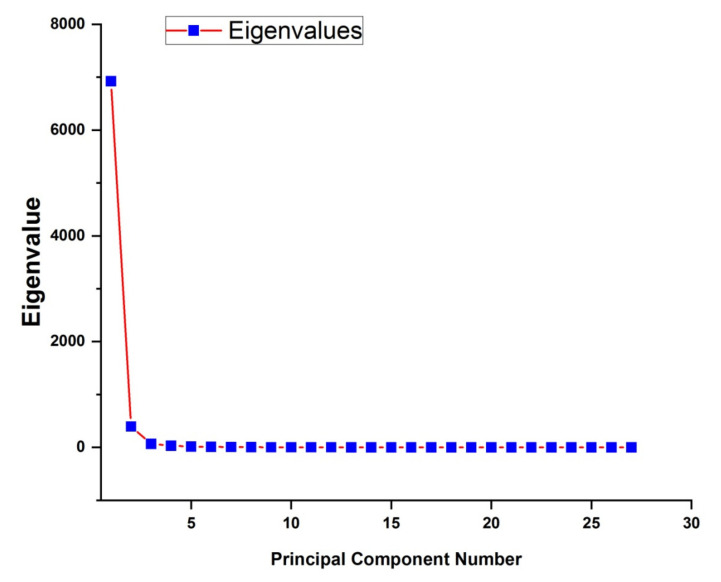
Scree plot obtained from PCA of FTIR spectra to figure out the required PCs in order to explain the variance of FTIR spectra of UHMWPE because of the addition of fillers and/or irradiations.

In order to visualize the data while keeping in view maximum possible % variance among the several sets, the transformation of data on new sets of axis is a useful tool. This set of new axis is called principal components (PCs) which enable the analytics to reduce the primary set of variables to new transformed PCs of significant variance. The first and foremost step is figuring out how many PCs are sufficient to unveil the hidden patterns/peaks/trends in the given data sets, and scree plot is used for this decision. A scree plot is a line plot of the Eigen values of each PCs in a multivariate study, and is used to decide about PCs that are required for further analysis. From the scree plot given in Figure 6, it is evident that three PCs are sufficient for explaining approximately 99.9% data set. It is worth mentioning that in problems where modifications at ppm level are required to be quantized, one cumulative variance > 99% needs to be considered for realistic analysis.

**Figure 7:**
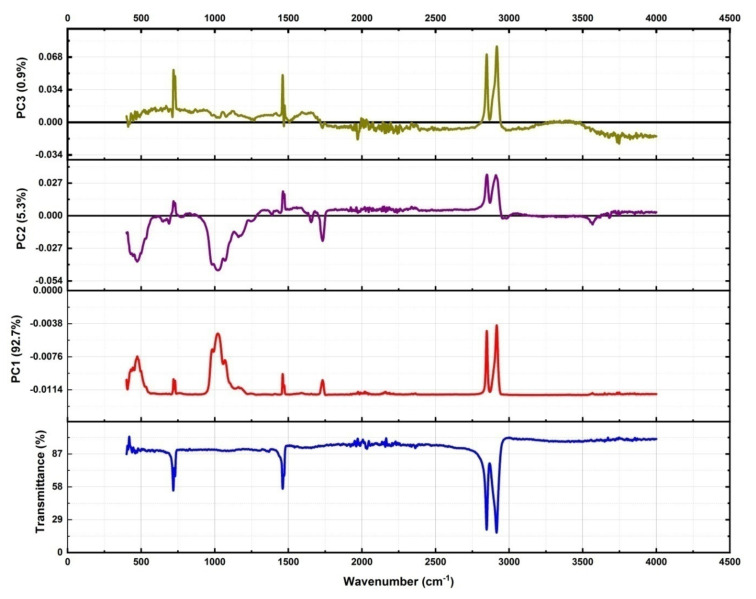
Factor loadings and reference plots for defining each PCs of interest with their corresponding percentage variance from reference FTIR spectra of UHMWPE.

Figure 6 indicates the plot obtained from PCA of FTIR spectra to figure out the required PCs in order to explain the variance of FTIR spectra of UHMWPE because of the addition of fillers and/or irradiations. Subsequent to the decision about the required numbers of PCs, next step is figuring out that how many numbers of factors are there for each PC and what are their strengths, i.e., figuring out factors and their loadings to corresponding PCs. It can be seen from Figure 7 that the corresponding factors for PC-1 are either polyethylene characteristics bands or the IR active region induced by fillers i.e., 440 cm^−1^, 456 cm^−1^, from 900–1200 cm^−1^, 1210 cm^−1^, 1596 cm^−1^. PC-2 unveils the modifications because of irradiation, e.g., gamma irradiations results in an increase of –CH_2_ stretching vibrations at 2849 and 2924 cm^−1^ [2,6,12,44], it is therefore, these two IR active bands have the positive loadings on PC-2 as shown in Figure 7. In addition to this, edged Mg–OH stretching vibrations are also quantified by PC-2. PC-3 quantifies the modifications in polyethylene characteristics bands i.e., CH_2_ rocking deformation, CH_2_ bending on additions of fillers particularly MWCNTs. In addition, this PC is also responsible for quantifying the peroxides and hydro peroxides as evident from Figure 7.

**Figure 8:**
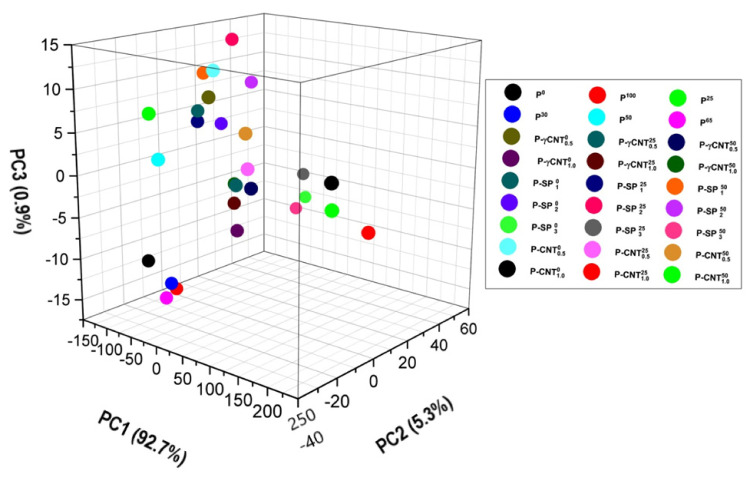
Score plotsof IR active bands on first three PCs to quantify the responsible factor (i.e., filler and/or irradiation) for inducing the variance in standard IR spectra of UHMWPE.

**Figure 9:**
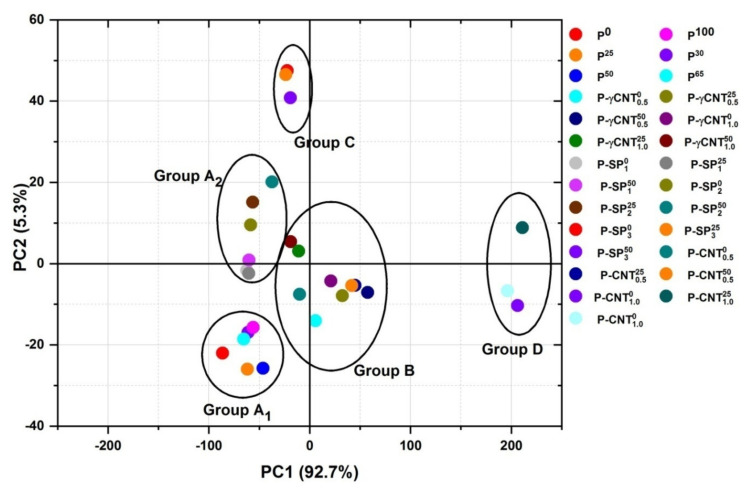
Score plots of IR active bands on first two PCs to quantify the responsible factor (i.e., filler and/or irradiation) for inducing the variance in standard IR spectra of UHMWPE.

The score plots for all the samples are shown in Figure 8 and Figure 9. It can be seen that all the samples are classified in four clusters i.e., A, B, C, and D, respectively. The two subgroups of A are encircled in Figure 9; A1 contains the pristine UHMWPE samples irradiated with 25, 30, 50, 65, and 100 kGy of gamma dose while the subgroup labeled as A2 contains the UHMWPE/Mg-silicate nano composites with 1.0% and 2.0% of Mg-silicate. Group C has members of UHMWPE/Mg-silicate composites with 3.0% of Mg-silicate by wt., and group D consists of UHMWPE/MWCNTs composites. Most of the members of group B are the UHMWPE/γMWCNTs nano composites.

The PC-1 is responsible for explaining approximately 93% of variance in data, this is because it corresponds either to polyethylene characteristics bands or the IR active region induced by fillers. The PC-2 is responsible for explaining approximately 5% of total variance, and PC-2 unveils the modifications because of irradiation, as evident from the loading plots shown in Figure 7. The IR active bands which are explained by the PC-2 also include the –C=O absorbance peak which is present at 1718 cm^−1^ as evident from the negative loadings of this PC with reference (transmittance) data as indicated in Figure 7. In addition to this, the increase or decrease of absorption corresponding to CH_2_ stretching vibrations, bending vibrations, and rocking deformations with respect to reference data is also explained by PC-2. Only the consideration of loading factor values and directions (+ve or −ve)] isrequired for choosing the suitable one from the given alternatives. Group D shows the larger variance in characteristics IR active band as evident from the strong positive loadings on PC-1, thus pointing to the fact that the concentration of the filler is higher in the elements of this group. The strong positive loadings of the members of Group C point toward the sever oxidation damage to the member of this group as compared to all other groups. The elements of group B are more particularly UHMWPE/γMWCNTs containing 1.0% of MWCNTs and are more close to origin, i.e., the loadings for these members on eachPC aresmall. However, irradiation of UHMWPE/γMWCNTs with gamma dose is responsible for a slight shift of the composite away from the origin along the diagonal of the 2nd quadrant. The diagonal of the 2nd quadrant indicated in Figure 9 shows a decrease in absorbance of filler’s IR active bands (in the region 900–1200 cm^−1^) and increase in absorbance of IR active bands associated to –CH_2_ bending vibrations of long chain polyethylene at 1460 and 1470 cm^−1^. This points toward the filler’s efficacy as free radical scavenger and enhancing the cross linking within the UHMWPE matrix because it is well established that radiation cross linking results in an increase in absorbance of CH_2_ bending vibrations of long chain polyethylene [12]. The score plot of PCA shown in Figure 8 and Figure 9 confirmed that for the group of irradiated sample, samples irradiated with 65 and 100 kGy are the best ones, and for the group of UHMWPE/Mg-silicate, sample with 2.0% of Mg-silicate and irradiated with 25 kGy of gamma dose is the optimum choice, and UHMWPE/γMWCNTs composites containing 1.0% of γMWCNTs and irradiated with 25 kGy are the better choice among the existing alternatives in the group.

**Figure 10:**
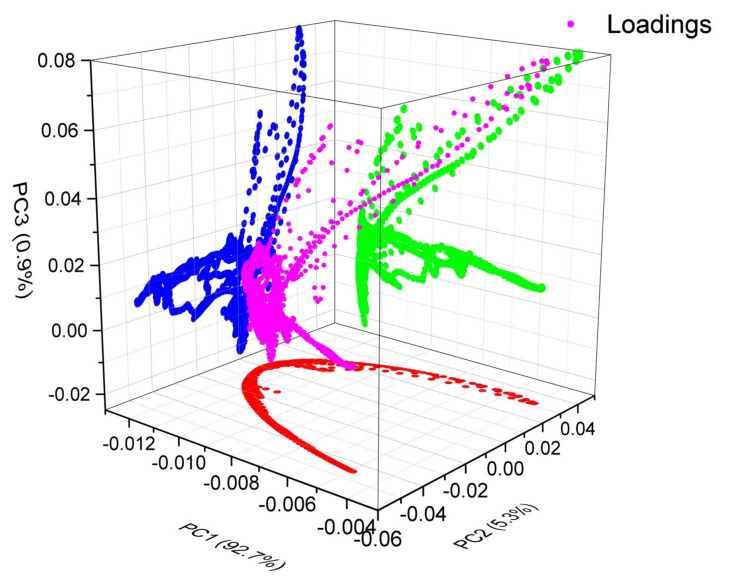
Loading plots on first three PCs to quantify the responsible factor (i.e., filler and/or irradiation) for inducing the variance in standard IR spectra of UHMWPE.

Figure 10 shows the loading plots on first three PCs to quantify the responsible factor (i.e., filler and/or irradiation) for inducing the variance in standard IR spectra of UHMWPE.

In summary the main theme of the work is using the concepts of variations in material attributes (IR active bands of UHMWPE here in this study) for selecting the optimal choice from existing alternatives. This can be done with multivariate analysis that can reveal the hidden pattern/factors that are responsible for inducing the variance in standard FTIR spectra of UHMWPE. Powerful multivariate statistical techniques including HCA, PCA have been used for finding the IR active vibration bands that are modified and/or induced because of gamma irradiating of UHMWPE, due to the incorporation of fillers, and due to the gamma irradiation of UHMWPE in the presence of pre-determined amount of fillers, respectively. PCA is a powerful technique that can reduced a large set of dispersed data into few significant dimensions of relevance importance (e.g., here in this study the alterations in IR active bands because of gamma irradiations, addition of fillers, and gamma irradiations in the presence of fillers are the main focus). This new set of dimensions of relevance importance are called as principal components (PCs), and each PC is responsible for explaining percentage variance in data from reference one because of its corresponding responsible factor. In the course of current study, PCA reduced the FTIR data of 26 samples in three PCs i.e., PC-1, PC-2, and PC-3, and quantified the structural modifications in UHMWPE because of the addition of fillers (Mg-Silicate or MWCNTs) and/or gamma treatment in terms of percentage variance from reference spectrum. FTIR spectra of UHMWPE is the reference and the other three PCs are responsible for explaining the total variance in the data due addition of fillers and irradiations. PC-1 which is responsible for explaining 92% of total variance corresponds to dispersion from reference data because of addition of fillers Mg-silicate; PC-2, which is responsible for explaining the 5.3% of total variance, corresponds to the spread of data from reference because of gamma irradiations; and PC-3, which covers the 0.9% of total variance, belongs to MWCNTs

## 4. Conclusions

FTIR spectroscopy in conjugation with chemometric analysis has been successfully employed to quantify the variations in the structure of UHMWPE because of the addition of fillers and/or gamma irradiations. The quantification has been done with respect to the fillers characteristics (concentrations and type) and/or absorbed gamma dose with the help of PCA combined with HCA. Moreover, the three PCs are sufficient enough to explain >99% variance in standard/reference data of UHMWPE in the course of this study where:

- PC-1 is found to be responsible for explaining the variance in polyethylene characteristics bands because of addition of fillers;
- PC-2 is responsible for explaining the variance in IR spectra because of radiation-induced oxidation and cross linking reactions;
- PC-3 quantifies the variance because of IR active bands of MWCNTs and the end products of oxidation reactions i.e., hydro peroxide regions.

Furthermore, PCA and HCA of FTIR data of all samples conclusively disclosed the 65 kGy irradiated sample as optimum choice from the group of irradiated samples. UHMWPE/Mg-silicate with 2.0% of Mg-silicate and irradiated with 25 kGy of gamma dose is the best one among the existing alternatives of UHMWPE composites, and UHMWPE composites containing 1.0% of γMWCNTs and irradiated with 25 kGy is the better choice among the existing alternatives of UHMWPE/MWCNTs.

## 5. Future Outlook

The presented results and methodology in this study can be used for assessing efficacy of fillers and free radical quencher in UHMWPE for resolving the long-term oxidation stability for orthopedic industrial applications with more reliable, authentic, simple, robust, and quantitatively efficient approach.

## Figures and Tables

**Table 1:** Identifications codes of samples used in the text of this paper.

Sample Code	UHMWPE Concentration by wt. (%)	Fillers Concentration by wt. (%)			Absorbed Dose (kGy)
		MWCNTs	γMWCNTS	Mg-Silicate	
P^0^	100	–	–	–	0
P^25^	100	–	–	–	25
P^30^	100	–	–	–	30
P^50^	100	–	–	–	50
P^65^	100	–	–	–	65
P^100^	100	–	–	–	100
P-γCNT_0.5_^0^	99.5	–	0.5	–	0
P-γCNT_0.5_^25^	99.5	–	0.5	–	25
P-γCNT_0.5_^50^	99.5	–	0.5	–	50
P-γCNT_1.0_^0^	99.0	–	1.0	–	0
P-γCNT_1.0_^25^	99.0	–	1.0	–	25
P-γCNT_1.0_^50^	99.0	–	1.0	–	50
P-CNT_0.5_^0^	99.5	0.5	–	–	0
P-CNT_0.5_^25^	99.5	0.5	–	–	25
P-CNT_0.5_^50^	99.5	0.5	–	–	50
P-CNT_1.0_^0^	99.0	1.0	–	–	0
P-CNT_1.0_^25^	99.0	1.0	–	–	25
P-CNT_1.0_^50^	99.0	1.0	–	–	50
P-SP_1_^0^	99.0	–	–	1.0	0
P-SP_1_^25^	99.0	–	–	1.0	25
P-SP_1_^50^	99.0	–	–	1.0	50
P-SP_2_^0^	98	–	–	2.0	0
P-SP_2_^25^	98	–	–	2.0	25
P-SP_2_^50^	98	–	–	2.0	50
P-SP_3_^0^	97	–	–	3.0	0
P-SP_3_^25^	97	–	–	3.0	25
P-SP_3_^50^	97	–	–	3.0	50

**Table 2:** Some IR active bands for subject matter of interest.

FTIR				Types and Origin of Vibrations
Band Position (cm^−1^)				
UHMWPE	UHMWPE Composites with MWCNTS		UHMWPE Hybrids with Magnesium Silicate	
	UHMWPE/MWCNTs	UHMWPE/γ-MWCNTs		
–	–	–	440	Si–O–Mg absorption band [43]
–	–	–	456	O–Si–O bending vibrations [43]
717 and 730	717 and 730	717 and 730	717 and 730	–CH_2_ long chain rocking–deformation [44]
910	910	910	910	Vinyl (–CH=CH_2_) [12]
963	963	963	963	*trans*-vinylene, (–CH=CH–) [12]
–	–	–	976, 1014, 1210	O–Si–O stretching vibrations [43]
–	–	–	1081	Si–O stretch and Siloxane linkage [12]
–	1100–900	1100–900	–	C–C stretching absorption [44]
–	1262	1262	–	C–O stretching absorption [44]
1305	1305	1305	1305	PE amorphous band [44]
–	1596	1596	–	–COO stretching absorption
1460 and 1470	1460 and 1470	1460 and 1470	1460 and 1470	CH_2_ bending vibrations [44]
1500–1700	1500–1700	1500–1700	1500–1700	C=C estimation area [36]
1650–1850	1650–1850	1650–1850	1650–1850	Carbonyl estimation band [36]
1896	1896	1896	1896	PE crystalline band
2849 and 2924	2849 and 2924	2849 and 2924	2849 and 2924	–CH_2_ stretching vibrations –
3000–3750	3000–3750	3000–3750	3000–3750	Peroxide bond area [36]
–	–	–	3536	Edged Mg–OH stretching [43]

## Data Availability

The data presented in this study are available on request from the corresponding author.
